# Incubating Isolated Mouse EDL Muscles with Creatine Improves Force Production and Twitch Kinetics in Fatigue Due to Reduction in Ionic Strength

**DOI:** 10.1371/journal.pone.0022742

**Published:** 2011-08-05

**Authors:** Stewart I. Head, Bronwen Greenaway, Stephen Chan

**Affiliations:** School of Medical Sciences, University of New South Wales, Sydney, New South Wales, Australia; University of Queensland, Australia

## Abstract

**Background:**

Creatine supplementation can improve performance during high intensity exercise in humans and improve muscle strength in certain myopathies. In this present study, we investigated the direct effects of acute creatine incubation on isolated mouse fast-twitch EDL muscles, and examined how these effects change with fatigue.

**Methods and Results:**

The extensor *digitorum longus* muscle from mice aged 12–14 weeks was isolated and stimulated with field electrodes to measure force characteristics in 3 different states: (i) before fatigue; (ii) immediately after a fatigue protocol; and (iii) after recovery. These served as the control measurements for the muscle. The muscle was then incubated in a creatine solution and washed. The measurement of force characteristics in the 3 different states was then repeated. In un-fatigued muscle, creatine incubation increased the maximal tetanic force. In fatigued muscle, creatine treatment increased the force produced at all frequencies of stimulation. Incubation also increased the rate of twitch relaxation and twitch contraction in fatigued muscle. During repetitive fatiguing stimulation, creatine-treated muscles took 55.1±9.5% longer than control muscles to lose half of their original force. Measurement of weight changes showed that creatine incubation increased EDL muscle mass by 7%.

**Conclusion:**

Acute creatine application improves force production in isolated fast-twitch EDL muscle, and these improvements are particularly apparent when the muscle is fatigued. One likely mechanism for this improvement is an increase in Ca^2+^ sensitivity of contractile proteins as a result of ionic strength decreases following creatine incubation.

## Introduction

The ingestion of supplementary creatine can increase intramuscular creatine and improve performance, particularly during maximally fatiguing exercise [1], [2]. It can also enhance muscle strength in certain myopathies, such as muscular dystrophy [3]. Creatine cannot be synthesized in skeletal muscle cells and is transported into skeletal muscle by a Na^+^-dependent transmembrane protein [4]. The creatine(Cr)/phosphocreatine(PCr) cycle acts as a temporal buffer of ATP during the first seconds of intense skeletal muscle contraction before glycolysis and mitochondrial mechanisms can respond to the increased demand for ATP. Without this buffering most skeletal muscles would run out of ATP after less than one second of intense exercise [5]. The Cr/PCr cycle is also an important source and transporter of ATP during the later stages of prolonged muscle activity. The Cr/PCr cycle is catalyzed by creatine kinase (CK), which has two main isoforms in skeletal muscle. Firstly, Mi-CK, the mitochondrial isoform [6], is bound to the inner membrane of the mitochondria [7] and is functionally linked to oxidative phosphorylation [8]. Secondly, MM-CK, the M-line isoform, is localised to the M-line of the A-band of the contractile apparatus and is functionally coupled to glycolysis [9]. This means that myosin ATPase preferentially uses ATP supplied by CK rather than cytosolic ATP [10]. This arrangement enables the Cr/PCr system to also function as a “spatial energy buffer” termed the “Creatine Phosphate Shuttle” [10], [11]. PCr and Cr “shuttle” molecules between the sites of ATP production and utilisation with several significant advantages. PCr and Cr have higher diffusion rates than ATP or ADP and therefore are more efficient as energy shuttles [12]. At the sarcomeres, where large amounts of ATP are hydrolyzed during repetitive contractions, the MM-CK allows for the immediate phosphorylation of ADP. This maintains a low ADP concentration, thus reducing the ADP-mediated leak of Ca^2+^ from the SR, which would reduce the releasable Ca^2+^ and hence reduce the force output of the muscle [13]. It also reduces the free inorganic phosphate (P_i_), slowing the entry of P_i_ into the SR where it precipitates the Ca^2+^ to cause a failure of Ca^2+^ release, reducing the force produced by the muscle [14]–[16]. Conversely, phosphorylation of Cr by Mi-CK keeps mitochondrial levels of ADP high, which stimulates the respiration rate and reduces the free energy required for ATP synthesis [8]. Mice which lack the MM-CK isoform of creatine kinase have been developed and display an inability to sustain maximal muscle force output during high intensity work [17]. Contractile kinetics are markedly slowed in the MM-CK-deficient mice [18]. Injection of small amounts of creatine into skeletal muscle deficient in all isoforms of creatine kinase restored its fatigue characteristics to wild-type specifications and interestingly also increases the Ca^2+^ concentration in the sarcoplasm [19], demonstrating that creatine can slow fatigue.

In fast-twitch muscles (muscles composed primarily of fast-twitch, or Type 2, fibres), repeated activation depletes stores of high energy phosphates (ATP and PCr) and there is a graded failure of Ca^2+^ release from the sarcoplasmic reticulum that is thought to be primarily responsible for the decline in the maximal force [15]. As mentioned above one mechanism causing this failure of Ca^2+^ release from the SR is the increase of P_i_ in the myoplasm (due to the increase in high energy phosphate utilisation). This P_i_ enters the SR and precipitates the free calcium as calcium phosphate [14], [15]. A reduction in P_i_ entering the SR may explain why fast-twitch muscles of creatine-kinase-deficient mice (all isoforms) are markedly more fatigue resistant than wild-types [20].

Creatine has also been reported to increase the Ca^2+^ sensitivity of the contractile proteins, by virtue of the reduction in ionic strength that occurs when water follows creatine osmotically into the muscle fibre [21]. The ability to produce more force for a given intracellular [Ca^2+^] would enhance muscle performance and alleviate some of the force loss resulting from reduced SR Ca^2+^ release during fatigue.

Despite some of the abovementioned benefits of creatine for muscle performance, several studies [22]–[25] have reported that oral creatine supplementation has no effect on either twitch or tetanic force in rodent muscles. As these studies used unpaired data sets they would not pick up small changes [21]. In addition they did not examine the effects of creatine supplementation on fatigued muscles. In a recent study, Bassit *et al.* [26] showed that in fatigued muscle the fall in twitch force was less pronounced in the creatine-supplemented group. Resistance to fatigue during 60 min of contractile activity was significantly greater in the creatine-supplemented group as compared to placebo.

The aim of our present study was to provide further insight into how creatine may enhance muscle performance, by examining the contractile properties of isolated whole muscles before and after the acute application of creatine, in the absence and presence of fatigue. We used an isolated fast-twitch muscle preparation, using the extensor digitorum longus (EDL) muscle from the mouse (composed almost entirely of fast glycolytic and fast oxidative fibres [27]), to examine the rate and extent of the force loss during muscle fatigue before and after incubating the muscle in creatine. In each case the muscle was used as its own control, with the muscle being fatigued in the absence of creatine, allowed to recover, then incubated in creatine, washed, and subjected again to the same fatigue protocol. This use of paired data [21] means that the statistics are very robust in being able to detect small differences as the same muscle is used for both treatment and control conditions; additionally, the topical application of creatine to the muscle removes the variability associated with oral creatine supplementation.

## Methods

### Ethics statement

Ethical approval was granted by the Animal Ethics Committee of the University of New South Wales (AEC 94\025). 12 mice (C57BL/10) aged 13 to 14 weeks were used. Animals were sacrificed using an overdose of Halothane (Fluothane™, Zeneca Limited, UK) followed by cervical dislocation.

### Water accumulation

In order to assess the water accumulation that resulted from incubation with 10 mM creatine, 7 EDL muscles were dissected out, blotted and weighed, then incubated in oxygenated Krebs solution containing 10 mM creatine for 30 minutes. They were then blotted on filter paper and weighed again. To see whether creatine incubation affects the diameter of muscle fibres, single enzymatically isolated flexor digitorum brevis (FDB) fibres were viewed under a Nikon inverted microscope at ×600, while perfused with oxygenated Krebs solution. A graticule was used to measure the fibre diameter at its widest point. The solution was then switched to one containing 10 mM creatine for 30 minutes, after which the fibres were observed during a washout period for 30 minutes. The isolated fibres were firmly attached to the glass coverslip and the graticule position was monitored throughout the procedure to ensure that its position relative to the fibre did not change during the procedure.

### Whole muscle setup and solutions

Details of the muscle set-up procedure have been reported elsewhere [28]. Briefly, the EDL muscles were removed and attached to a force transducer and positioned between two platinum plates so that the muscle could be stimulated electrically and the resultant force response recorded. The muscles were set to their optimal length and were continuously superfused with Krebs solution (in mM): 4.75 KCl, 118 NaCl, 1.18 KH_2_PO_4_, 1.18 MgSO_4_, 24.8 NaHCO_3_, 2.5 CaCl_2_, 11 glucose, bubbled with 95% O_2_ 5% CO_2_ to maintain the pH at 7.4. The optimal length was termed *L*_0_ and all subsequent measures were made at this muscle length. After determining the set length of the muscle it was allowed to equilibrate for 5 minutes in the bath.

Following this a force-frequency curve was generated using frequencies of 2, 5, 10, 15, 20, 25, 30, 50, 60, 75 and 100 Hz. This served as the “pre-fatigue” force-frequency curve for the muscle. A fatigue run was then carried out using our published fatigue protocol [29], [30], stimulating the muscle at 100 Hz, 1 second on, 1 second off for 30 seconds. This protocol typically reduced the force by 70–80%. Immediately following this, a force-frequency curve was generated to measure the extent and nature of fatigue. This served as the “fatigue” force-frequency curve. A 20-minute recovery period followed in which the muscle was stimulated for 200 msec at 100 Hz once every 5 minutes in order to monitor the rate of recovery from fatigue. A force-frequency curve was then generated to determine recovery. This served as the “recovery” force-frequency curve. In our laboratory, we have observed that EDL muscles can be run up to five times through the protocol described above, without any significant deterioration of the muscle (unpublished data).

Following determination of the force-frequency characteristics of untreated muscle (the control condition), the muscle was incubated in a 9.9 mM creatine solution, prepared by dissolving creatine (Creatine Anhydrous from SIGMA) in the Krebs solution described above. The incubation period was 30 minutes and the muscle was stimulated at 100 Hz for 200 msec once every five minutes. The creatine-specific transporter protein CreaT [31] controls cellular creatine uptake. This moves creatine into the cell, against a concentration gradient, in a sodium- and chloride-dependent process [31]. Stimulating the muscle throughout incubation facilitates this uptake as described by Odoom *et al.* [32]. The rate of creatine uptake into the muscle under these conditions is estimated to be 70 nmol gww^−1^ [4]. The solution containing creatine was then washed out over a 5-min period and the muscle re-perfused with Krebs solution, so that the force produced by the muscle was not secondarily affected by having creatine present in the bathing solutions during the experimental procedures. The protocol from the pre-fatigue force-frequency curve to the post-fatigue force-frequency curve was then repeated. All experiments were undertaken at 22°C.

In the presentation of results, all forces are expressed as specific force (absolute force divided by cross-sectional area). The mean cross-sectional area of each muscle was calculated by dividing the muscle's wet mass by the product of its optimum length and the density of mammalian skeletal muscle (1.06 g cm^−3^). The wet muscle mass was measured at the end of each experiment and hence represents the muscle mass following creatine incubation. In the water accumulation experiments described above, it was found that creatine incubation resulted in an average 7.3% increase in muscle weight. To allow for this, cross-sectional areas have been reduced by this amount in calculating specific forces before creatine incubation.

### Statistical tests and analysis

A two-tailed paired *t*-test (GraphPad Prism) was used to test the significance of treatments. The significance level was *P*<0.05. However, when comparing control force versus creatine-treated force at each stimulation frequency of the force-frequency curve, a different test was used to reduce the possibility of Type I error arising from the large number of comparisons. Here we used Bonferroni multiple comparison post-tests following two-way repeated measures ANOVA, with an overall significance level of 5% (GraphPad Prism).

One further analysis performed on the force-frequency data was to fit a sigmoidal curve to the forces measured in every force-frequency activation sequence. This curve relates the muscle force *P* to the stimulation frequency *f* according to the following equation [33]:
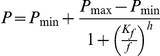
where *P*_max_ is the maximum force, *P*_min_ is the minimum force, *K_f_* is the half-frequency (the frequency at which force is halfway between minimum and maximum) and *h* is the Hill coefficient (an indication of the steepness of the curve).

## Results

### Whole muscle mass and single fibre diameter in 10 mM creatine

**Figure 1:**
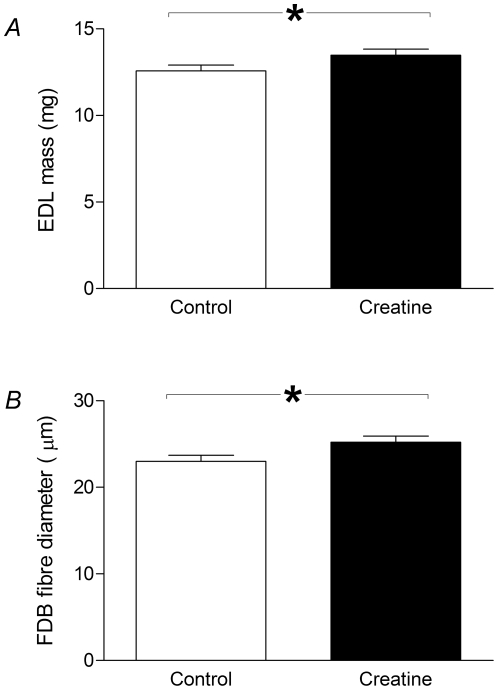
Changes in EDL muscle mass and FDB fibre diameter following creatine incubation. Creatine incubation resulted in a significant increase in the wet weight of whole EDL muscle (*A*) and a significant increase in the diameter of single FDB fibres (*B*), demonstrating the uptake of creatine by these muscles and fibres. Statistically significant differences are indicated by “*” (*P*<0.05).

Creatine enters skeletal muscle by a saturable process, with an accompanying increase in intracellular water that is evidenced by an increase in muscle weight [34]. To show that our incubation procedure results in the successful uptake of creatine, we incubated EDL muscles and FDB fibres for 30 minutes in oxygenated Krebs solution containing 10 mM creatine, and measured resulting changes in muscle weight and fibre diameter. Following incubation, there was an increase in EDL wet weight of 7% (Fig. 1*A*), and an increase in FDB fibre diameter of 9% (Fig. 1*B*). These results demonstrate that our incubation procedure results in the uptake of creatine by the muscles and by the individual fibres [34].

### Force-frequency curves from EDL muscles

**Figure 2:**
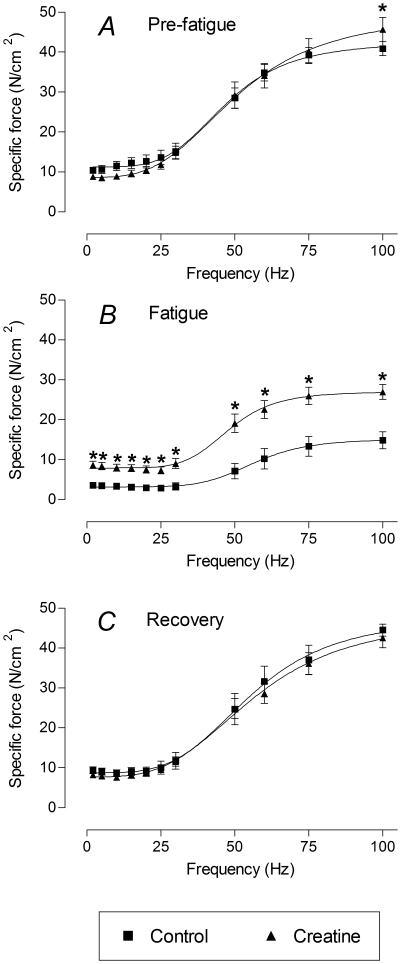
Force-frequency curves for EDL muscles before and after creatine treatment. Each graph shows the force-frequency curves under control and creatine-treated conditions. In the pre-fatigue state (*A*), creatine significantly increased the force produced at 100 Hz. In the fatigued state (*B*), creatine incubation significantly increased the force produced at every frequency. In the recovered state (*C*), creatine had no significant effect on the force at any frequency. Statistically significant differences are indicated by “*” (*P*<0.05). Tests for significance at each frequency were conducted using Bonferroni multiple comparison post-tests following two-way repeated measures ANOVA (GraphPad Prism).

The effects of creatine incubation on the force-frequency curves are illustrated in Fig. 2. Before fatigue (Fig. 2*A*), creatine treatment resulted in a significantly higher force at 100 Hz. In fatigued muscle (Fig. 2*B*), incubation in a creatine load solution prevented a significant amount of the loss of force seen as a result of repetitive activation in the same muscle under control (untreated) conditions. In the fatigued state, the creatine-treated muscle was found to produce significantly more force at all frequencies when compared with the muscle before creatine incubation (Fig. 2*B*). Maximal tetanic force in fatigued muscle was 27.0±1.85 N cm^−2^ following creatine incubation compared with 14.9±2.13 N cm^−2^ before creatine treatment (Fig. 2*B*). After recovery from fatigue (Fig. 2*C*), creatine treatment did not significantly affect the force at any frequency when compared with control.

**Figure 3:**
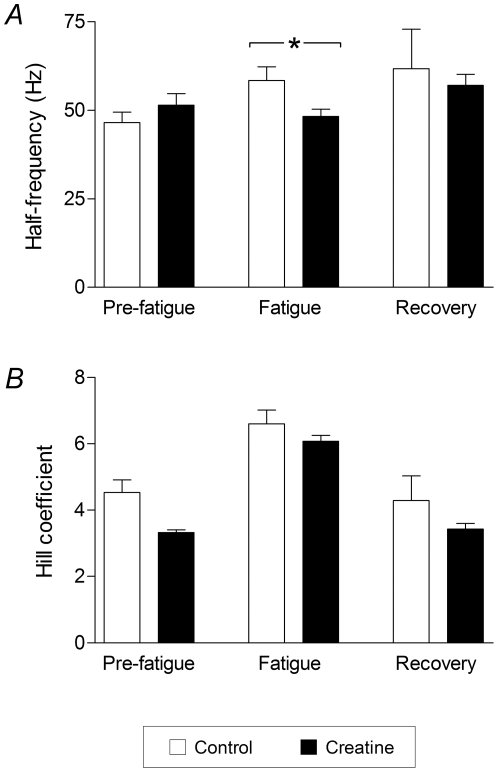
Half-frequency and Hill coefficient of force-frequency curves. Sigmoidal curves were fitted to the force-frequency data to obtain the half-frequency (*A*) and Hill coefficient (*B*). Creatine treatment resulted in a significant reduction in the half-frequency in fatigued muscle. Statistically significant differences are indicated by “*” (*P*<0.05).

Sigmoidal curves were fitted to the force-frequency data to obtain the half-frequency (the frequency at which force is halfway between minimum and maximum) and the Hill coefficient (a measure of the steepness of the curve). The values of these parameters are shown in Fig. 3. When the muscle was fatigued, creatine incubation resulted in a significant reduction of the half-frequency (Fig. 3*A*). In terms of the force-frequency curves for fatigued muscle shown in Fig. 2*B*, the curve for creatine-treated muscle has shifted to the left by 10.2 Hz compared to the control curve. No significant differences in the Hill coefficient (steepness of the curve) were found between creatine-treated and control conditions (Fig. 3*B*).

### Twitch and tetanus in EDL muscles

**Figure 4:**
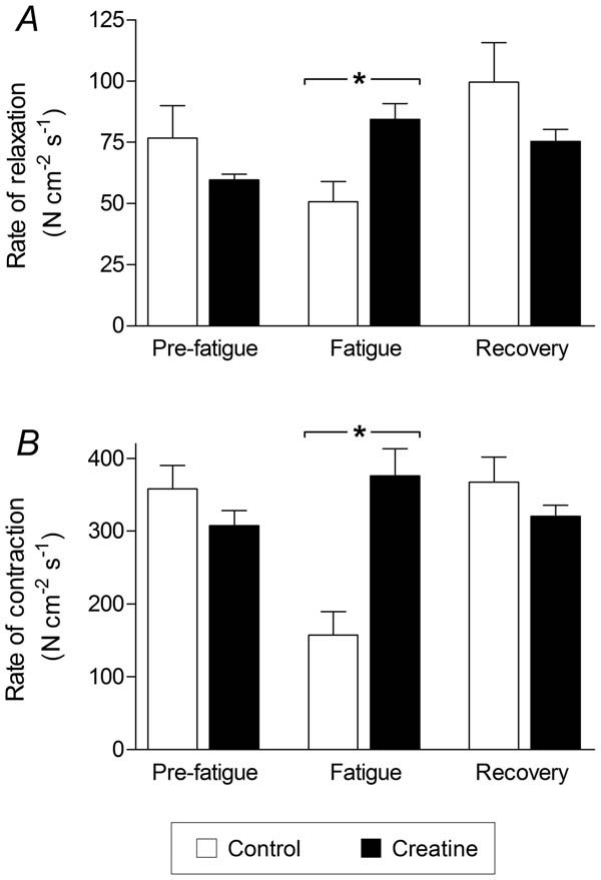
Twitch kinetics of EDL muscles before and after creatine treatment. *A*, Rate of relaxation. Creatine treatment resulted in significantly faster relaxation in fatigued muscle. *B*, Rate of contraction. Creatine treatment resulted in significantly faster contraction in fatigued muscle. Statistically significant differences are indicated by “*” (*P*<0.05).

The rate of relaxation during a muscle twitch, calculated as the fall in force divided by the change in time during the first 50% of relaxation, is shown in Fig. 4*A*. In fatigued muscle, the rate of relaxation following creatine incubation was significantly faster than control. The rate of contraction during a muscle twitch, calculated as the peak twitch force divided by the time taken to reach peak force, is shown in Fig. 4*B*. In fatigue, the rate of contraction following creatine incubation was significantly faster than control.

**Figure 5:**
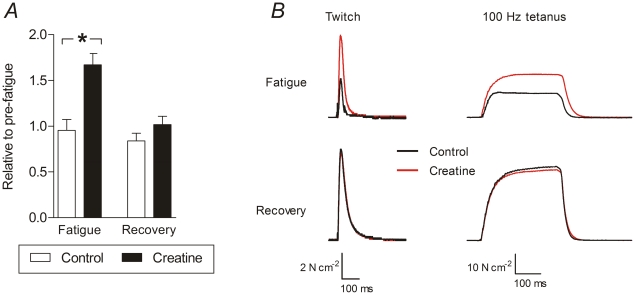
Twitch and tetanus in EDL muscles before and after creatine treatment. *A*, twitch to tetanus ratios expressed relative to the pre-fatigue twitch to tetanus ratio. Before creatine incubation, the twitch to tetanus ratio in fatigued muscle was similar to that in the pre-fatigue state. After creatine incubation however, the twitch to tetanus ratio in the fatigued state was significantly higher than in the pre-fatigue state. Statistically significant differences are indicated by “*” (*P*<0.05). *B*, force tracings of twitches and 100-Hz tetani obtained from one EDL muscle. In the fatigued muscle, creatine incubation increases the twitch force and tetanic force. In the recovered muscle however, creatine incubation makes little difference to the twitch and tetanic forces.

The twitch-to-tetanus ratio was unchanged for the creatine-treated condition with respect to the control condition both before fatigue and after recovery. In creatine-treated muscles, however, there was a difference between the ratio in fatigue and the ratios for the pre-fatigue and recovered states. Notably this difference did not occur before creatine treatment. Fig. 5*A* shows that creatine treatment leads to a comparatively greater increase in twitch to tetanus ratio when the EDL muscles are fatigued compared to controls.

Fig. 5*B* shows that creatine treatment increases twitch and tetanic force when the muscle is fatigued, but has little effect on twitch and tetanic force when the muscle is recovered.

### Rate of fatigue in EDL muscles

**Figure 6:**
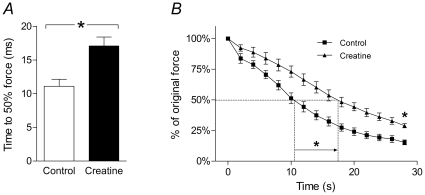
Fatigue run in EDL muscles before and after creatine treatment. *A*, time taken for force to fall to 50% of original force. Creatine incubation resulted in a significant increase in this parameter. *B*, pattern of force decline during the fatigue run, showing the effect of creatine treatment in improving the resistance of muscles to fatigue. It is evident that creatine incubation is associated with an increase in the time taken for force to fall to 50%, as well as a reduction in the total force decline over the whole fatigue run. Statistically significant differences are indicated by “*” (*P*<0.05).

Creatine significantly increased the EDL muscle's resistance to fatigue. The parameter measured during the fatigue runs was the time taken for the maximum force produced by the muscle to fall to 50% of the pre-fatigue maximum. Creatine-treated muscle took 55.1±9.5% (*P*<0.05) longer than the control for the force to fall to this point (Fig. 6*A*). Total force decline, as a percentage of initial force, was 84.8±2.0% before creatine incubation and 71.1±1.8% after creatine incubation (*P*<0.05). Fig. 6*B* shows the pattern of force decline during the fatigue run, before and after creatine incubation.

## Discussion

### Creatine and its effects on ionic strength and force production

In the present study we have shown that acute application of creatine enhances force production in isolated fast-twitch EDL muscle, especially during fatigue. In unfatigued muscle, creatine enhanced maximum force production (Fig. 2*A*). In fatigued muscle, creatine reduced the half-frequency (Fig. 3*A*), resulting in a left-shifting of the force-frequency relationship and an enhanced force production at every stimulation frequency (Fig. 2*B*).

We have also shown that acute incubation with creatine increased the weight of intact muscles and increased the diameter of individual fibres (Fig. 1). The most likely mechanism for this is that the uptake of creatine is accompanied by an increase in cellular water which occurs to maintain osmotic balance [35]. Increases in muscle mass along with water retention have frequently been reported as a side effect of creatine ingestion. Studies [36]–[38] reported increases in fat-free body mass of 1–2 kg which equates with a 7% increase in muscle water in an average human [21]. This is consistent with the 7% increase we observed in the mass of whole muscles following creatine incubation (Fig. 1*A*).

Our observed effects of creatine on force and muscle mass are consistent with the study of Murphy *et al.* [21], who found that increased creatine concentration, accompanied by a reduction in ionic strength mimicking the osmotic uptake of water, enhanced the Ca^2+^ sensitivity and maximum Ca^2+^-activated force in mechanically skinned EDL fibres. Under these conditions, the force-pCa curve for EDL fibres was shifted to the left [21], which is analogous to the left-shifting of the force-frequency curve caused by creatine in our fatigued muscles. Hence an increase in the Ca^2+^ sensitivity of the contractile proteins could underlie the shift of the force-frequency curve that we found when creatine was applied to fatigued muscle.

Murphy *et al.* [21] concluded that the beneficial effects of creatine occurred through the reduction in ionic strength, rather than some direct action of creatine, because creatine actually had an inhibitory effect on force and Ca^2+^ sensitivity when ionic strength was left unchanged. In our present study, we can assume that the ionic strength within our EDL muscles has decreased, since the increase in mass following creatine incubation suggests osmotic water uptake. Hence we postulate that a reduction in ionic strength following creatine incubation is responsible for the enhanced force production of our creatine-treated muscles. The influence of ionic strength on muscle contractility is an established observation in the literature [39]–[41]; it is possible that a reduction in ionic strength enhances the force production and Ca^2+^ sensitivity of the contractile apparatus because of reduced competition from monovalent cations such as K^+^ and Na^+^ for binding sites on the thin-filament regulatory proteins [41].

### Creatine and fatiguing stimulation

Fatigue can be defined as the inability of a muscle to maintain maximal force production as a result of repeated activity. Failure of Ca^2+^ release from the SR is considered to be a major cause of fatigue [42], [43]. Fatigued muscle also suffers in respect of contractile kinetics and there is a slowing of contraction and relaxation. Using the alteration of these parameters as a measure of fatigue a major finding of this study was that incubation in a creatine solution, followed by a wash before initiating the fatigue protocol, caused an improved resistance to fatigue in mouse fast-twitch EDL muscle (Fig. 6).

Why does creatine have this inhibitory effect on fatigue? One mechanism that can be deduced from earlier studies is that creatine helps to maintain Ca^2+^ release during fatigue. When creatine is transported into the muscle fibre a proportion of it will be phosphorylated and become part of the phosphocreatine cycle [44]. The Cr/PCr cycle is the major provider of ATP during intense activity in skeletal muscle. The failure of Ca^2+^ release is thought to be primarily due to the build up of P_i_ which occurs due to the high rates of ATP hydrolysis during intense muscle activation. This P_i_ enters the SR via an ATP-dependent ion channel transport mechanism (the channel is activated by low ATP) where it precipitates the free Ca^2+^ resulting in a failure of Ca^2+^ release [42]. Thus increased levels of PCr will keep the P_i_ levels lower by using the free inorganic phosphate to regenerate ATP from ADP. In addition the open probability of the phosphate transporter in the SR will be reduced as ATP levels are higher. Supporting this, Gallo *et al.* [45] noted that high levels of creatine supplementation which increased intramuscular creatine by 20% improved fatigue resistance in rat EDL muscle by 20% and 7% after 10 and 30 s of stimulation, respectively.

However, another mechanism has been proposed where force is enhanced due to the increased Ca^2+^ sensitivity of the contractile proteins arising from the reduction in ionic strength that accompanies Cr loading [21]. This is a likely mechanism in our current study, given the left-shifting of the force-frequency curve in fatigue following creatine incubation. Thus even if Ca^2+^ release from the SR was impaired during fatigue, creatine incubation may attenuate the resulting loss of force by making the contractile proteins more sensitive to Ca^2+^.

### Creatine and its effect on twitch kinetics

The slowing of contractile kinetics during fatigue is a well established finding in the literature. The decreased speed of relaxation [46] and contraction [47] becomes progressively more pronounced throughout a fatiguing protocol. Relaxation processes account for an important fraction of total energy consumption in muscles during short repeated contractions [48], [49]. Relaxation is a multi-faceted process and different techniques for measurement of changes to the relaxation process have been used. In a review by Gillis [50], relaxation is described as having two distinct phases. The first is the plateau which is a slow linear decline in tension with no change in muscle length. During this phase the intracellular [Ca^2+^] falls rapidly [51]. In the second phase tension drops rapidly with Ca^2+^ levels relatively constant [51]. Creatine supplementation has been shown to decrease half relaxation time [52] and to improve Ca^2+^ handling in dystrophic muscle [31]. The creatine analogue β-GPA (β-Guanidinopropionoic acid) depletes cellular creatine (and hence phosophocreatine levels) and causes an increase in time to half relaxation [53]. In the absence of the phosphocreatine buffering system (achieved by inhibiting creatine kinase using dinitroflourobenzene) shortening velocity can be up to 30% slower than the control both in mouse skeletal muscle [47] and in frog skeletal muscle [54]. Conversely, mice genetically modified to have an increased expression of creatine kinase demonstrated a faster shortening velocity during a 5 second tetanus [55]. Steeghs *et al.* [56] found that CK-deficient muscle tested *in vivo* displayed an impaired peak tetanic force, a reduced ability to sustain maximal contractions and an increased time to half relaxation. Further, in myotube cultures from the same strain of mouse, both the release and sequestration of Ca^2+^ were negatively affected as measured by a reduced peak and an extended duration of the Ca^2+^ released in a twitch.

In the present study it is possible that the increased rates of contraction and relaxation seen in fatigued muscle pre-incubated in creatine (Fig. 4) is a consequence of lowered ionic strength. However, we cannot rule out an increased supply of PCr as a result of creatine uptake in improving the contractile kinetics. In humans, creatine supplementation increased intramuscular creatine by 8% and significantly improved performance during the transition from rest to both moderate- and heavy-intensity exercise, and also improved performance in the transition from moderate-intensity exercise to rest [57].

### Conclusions

We have shown that acute incubation with creatine enhances force production in fast-twitch EDL muscle, especially when the muscle is fatigued. Such an effect is likely to occur through an increased Ca^2+^ sensitivity of the contractile proteins due to the reduction in ionic strength that accompanies creatine incubation. The improved resistance to loss of force during fatiguing stimulation is also likely to be explained by an increased Ca^2+^ sensitivity. We have also shown that in fatigued muscle, creatine incubation increases the rate of twitch contraction and relaxation. In fatigued muscle, even small changes in force production and contractile kinetics could be very important in terms of human performance in a race situation. Thus the effects of creatine incubation on isolated muscle demonstrated in our study provide insight into the improved muscle performance observed following creatine supplementation in humans. Providing there are no as yet undiscovered health risks associated with creatine supplementation, creatine may be of great benefit both to athletes and as a therapeutic tool for the treatment of neuromuscular disorders.
